# Vascular and Platelet Effects of Tomato Soffritto Intake in Overweight and Obese Subjects

**DOI:** 10.3390/nu15245084

**Published:** 2023-12-12

**Authors:** Anallely López-Yerena, Teresa Padro, Victoria de Santisteban Villaplana, Natàlia Muñoz-García, Antonio Pérez, Gemma Vilahur, Lina Badimon

**Affiliations:** 1 Cardiovascular Program-ICCC, Institut d’Investigació Biomèdica Sant Pau (IIB SANT PAU), 08041 Barcelona, Spain; naye.yerena@gmail.com (A.L.-Y.); tpadro@santpau.cat (T.P.); vsantisteban@santpau.cat (V.d.S.V.); nmunoz@santpau.cat (N.M.-G.); gvilahur@santpau.cat (G.V.); 2 Centro de Investigación Biomédica en Red Cardiovascular (CIBER-CV), Instituto de Salud Carlos III, 28029 Madrid, Spain; 3 Faculty of Pharmacy, University of Barcelona, 08036 Barcelona, Spain; 4 Servicio de Endocrinología y Nutrición, Hospital de la Santa Creu i Sant Pau, IIB Sant Pau, Universitat Autònoma de Barcelona, 08041 Barcelona, Spain; aperez@santpau.cat; 5 CIBER de Diabetes y Enfermedades Metabólicas Asociadas (CIBERDEM), 08041 Barcelona, Spain; 6 Cardiovascular Research Chair, Universitat Autònoma de Barcelona (UAB), 08193 Barcelona, Spain

**Keywords:** dietary antiplatelet, lipid profile, endothelial activation, cardiovascular disease, Mediterranean diet

## Abstract

Tomatoes are known for their numerous health benefits, including antioxidants, anti-cancer, antimicrobial, anti-inflammatory, anti-neurodegenerative, antiplatelet, and cardio-protective properties. However, their potential health benefits in the Mediterranean diet’s popular soffritto remain largely unexplored in scientific research. The objective was to evaluate the effects of soffritto intake on platelet activity, vascular endothelial function, weight, lipid profile, and blood parameters. In a prospective, controlled, randomized two-arm longitudinal cross-over trial, 40 overweight and obese individuals received 100 g/day of soffritto, or a control, for 42 days. The primary outcome was the effect on vascular endothelial function and platelet activity. As exploratory secondary outcomes, anthropometric measures, serum lipid profile, and hemogram profile were measured before and after a 6-week intervention with or without soffritto supplementation. Compared with the control group, soffritto supplementation for six weeks improved collagen-induced (−5.10 ± 3.06%) platelet aggregation (*p* < 0.05). In addition, after six weeks, a reduction in ADP-induced aggregation (−3.67 ± 1.68%) was also only observed in the soffritto group (*p* < 0.05). No significant effects of the soffritto intake were observed on vascular endothelial function, anthropometric measures, serum lipid profile, or blood parameters (*p* > 0.05). In conclusion, as a basic culinary technique, soffritto may have a role in the primary prevention of cardiovascular disease by reducing platelet activation, which could contribute to a reduction in thrombotic events.

## 1. Introduction

The occurrence of overweight and obesity is persistently rising in both developed and developing countries [1,2]. According to the Food and Drug Organizations of the United Nations (FAO), overweight and obesity are leading risks for global deaths [3]. Each year, roughly 3.4 million adults lose their lives due to complications arising from being overweight or obese. In addition, 44% of the diabetes burden, 23% of the ischemic heart disease burden, and between 7 and 41% of certain cancer burdens are attributable to overweight and obesity. Obesity is a persistent inflammatory state characterized by elevated body fat levels, resulting in an augmentation of inflammatory mediators circulating in the body [4], systemic oxidative stress [5], and an attenuation of antioxidant status [6]. Extracellular vesicles (EVs) are a heterogeneous group of small membrane-bound particles that are released into the circulation by many different cell types when activated or apoptotic. The potential use of circulating EVs (cEVs) as biomarkers of cellular damage in cardiovascular disease (CVD) has been studied for years due to their high content in the molecules of the parental cells from which they originate. They also play a role in intercellular communication and intricate regulatory pathways, given their ability to transport various molecules within the same lipid envelope [7].

Tomato, a staple of the Mediterranean diet, is an important source of antioxidants, mainly carotenoids (such as lycopene), phenolic compounds, and vitamins [8,9,10]. These fruits can be consumed either fresh or processed, and given their nature as bioactive compounds, they have been considered a strategy to combat chronic inflammatory conditions in overweight and obesity. In a randomized controlled clinical trial with overweight and obese females was demonstrated that daily intake of tomato juice (330 mL, 20 days) leads a reduction in inflammation [4], oxidative stress mainly in overweight population [11] and no effect on antioxidant enzyme activity and total antioxidant capacity [6]. In a study using an animal model was evidenced the anti-inflammatory effect of lycopene [12]. In addition, a protection against vascular alteration was demonstrated for tomato-based soffritto (rich in carotenoids and polyphenols) in obese rats [13]. Opposed to these positive results, high daily consumption of tomato-based products (32–50 mg lycopene/day) or lycopene supplements (10 mg/day) was ineffective in reducing conventional CVD risk markers in moderately overweight, healthy, middle-aged individuals [14]. Additionally, two studies demonstrated that the intake of tomato-based products did not induce changes in blood hemogram profile in healthy populations [15,16], but little is known about the effect in obese populations. Other cardiovascular health benefits such as inhibition of platelet aggregation have also been at-tributed to the intake of tomato or tomato-based products. The antiplatelet aggregation effect was demonstrated in healthy humans after water-soluble tomato extract [17] and tomato extract [18,19]. However, a lingering research gap pertains to the impact of soffritto consumption on the inhibition of platelet aggregation in populations with obesity and overweight.

Discrepancies in the beneficial health effect of tomato or tomato-based products consumption have been related to the bioavailability of lycopene, as it is higher in tomato paste than in fresh tomatoes [20]. Regarding home-cooking techniques for tomatoes, it has been shown that the availability of bioactive compounds (carotenoids and phenolic compounds) is improved during cooking, such as in the case of soffritto [21,22]. These studies demonstrated that the presence of olive oil enhanced the bioaccessibility of certain polyphenols in tomatoes, and the use of onions during the cooking process effectively protect-ed them from oxidation [21,22]. Although it is also well known that tomato sautéing con-tributes to increase the content of bioactive compounds such as polyphenols [23], so far little is known about its potential health effect on vulnerable populations such as those who are overweight and obese. Hence, we designed a clinical study aimed at investigating the effects of a daily intake of soffritto in overweight or obese class 1 individuals without other cardiovascular risk factors such as dyslipidemia, type 2 diabetes, or hypertension, focusing on platelet activity. Additionally, vascular endothelial function, weight changes, lipoprotein profile, and arterial stiffness were also assessed to discriminate against any undesired effects after daily soffritto intake.

## 2. Materials and Methods

### 2.1. Subjects

Forty healthy adult men (*n* = 27) and women (*n* = 13) with ages ranging from 27 to 60 years, non-smokers, and overweight (BMI: 25.0–29.9 kg/m^2^) or obese class 1 (BMI: 30–34.9 kg/m^2^) were invited to participate in the study through word of mouth and a newspaper advertisement. Subjects were excluded if they reported existing chronic illnesses including cancer, diabetes mellitus, dyslipidemia, hypertension, liver, heart, or kidney disease (creatinine > 2 mg/dL) and excessive alcohol consumption (>60 g/day of ethanol). Additional exclusion criteria comprised the usage of lipid-lowering medications, β-blockers, or diuretics; a prior history of CVD or psychiatric disorders; the administration of psychotropic medications; being in a weight-loss program; or intolerance to tomato-based products. In addition, the use of aspirin and other platelet inhibitors was also an exclusion criterion. To confirm their health status, all subjects underwent a complete physical examination conducted by the study physician before inclusion.

### 2.2. Ethics Statement

The entire participant signed an informed consent and was able to withdraw from the study at any time without giving a reason. This study received approval from the Human Ethical Review Committee of Hospital Santa Creu I Sant Pau (Barcelona, Spain), with the reference number 12/181 and the date of approval being 11 January 2013.

### 2.3. Soffritto Samples

Soffritto samples were supplied by PREPARADOS ALIMENTICIOS S.A. and consisted of a cooked mix of tomato, onion, extra virgin olive oil, sugar, and salt (see details of product composition in Table 1). Each sample was enclosed in a glass container containing 100 g of soffritto. Soffritto includes various carotenoids such as lycopene, lutein, α-carotene, and β-carotene. The manufacturer previously utilized high-performance liquid chromatography to assess the lycopene levels in the soffritto, revealing a content of 215.57 mg/kg. This measurement encompassed various isomeric forms, including 5-*cis*-, 9-*cis*-, 13-*cis*-lycopene, and *trans*-lycopene. Additional bioactive constituents found in soffritto included an oxygen radical absorbance capacity of 2891.09 mm Trolox equivalents per 100 g of quercetin, glycosylated quercetin, rutin, and ascorbic acid (at 5.51, 37.22, 91.51, and 73.3 mg/kg, respectively).

### 2.4. Study Design and Dietary Monitoring

**Figure 1:**
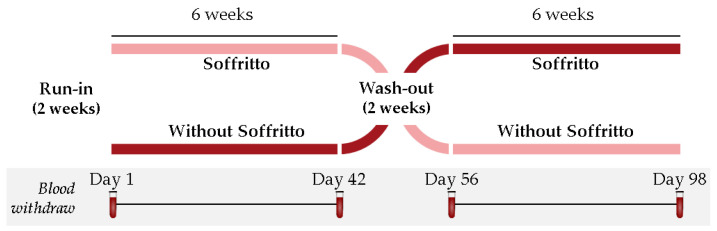
Flow diagram describing the study design.

The study carried out was a prospective, controlled, randomized two-arm longitudinal crossover trial [Clinical Trial: NCT06161883], performed in a single center, as indicated in Figure 1.

After a run-in period of two weeks, the study was carried out for 14 weeks, including two sequential intervention periods (6 weeks each). In the first intervention period, participants were randomly separated into two different arms (*n* = 20/arm), in which volunteers were administered a soffritto (100 g/day) on top of their habitual diet or a control group without a soffritto. After the first six-week period, participants had a wash-out phase of two weeks, followed by a second six-week period in which groups exchanged their interventions. The selection of the wash-out period was based on the bioavailability of lycopene in plasma [24]. A simple randomization was performed using computer-generated random numbers. A random number and group were assigned to each subject at the moment of enrollment, purely by chance.

During the duration of the study, the volunteers were asked to maintain their usual diet, excluding raw or cooked tomatoes as well as tomato-based products (sauces, ketchup, juices, etc.) other than those administered during the study in the corresponding periods. Dietary patterns, assessed using food frequency questionnaires, were documented before each visit, and minimal alterations in dietary habits were reported. Adherence was monitored through regular telephone communication with participants, coupled with interviews conducted at the conclusion of each intervention period. Volunteers also recorded whether they had consumed tomato or tomato-based products on a diary card each day. Additionally, a clinician evaluated potential side effects or symptoms, such as flushing, bloating, dizziness, vomiting, and diarrhea, which could be linked to soffritto intake, at the conclusion of each intervention period. However, no adverse effects were observed in the volunteers.

Blood samples, obtained following a twelve-hour fasting period, were collected on days 1 and 42 to establish baseline and first treatment period endpoints. Similarly, blood samples were obtained on days 56 and 98, representing baseline and second treatment period endpoints (Figure 1). Blood samples were collected without or with an anticoagulant (citrate or EDTA); separation was accomplished through centrifugation, aliquoted, and stored at −80 °C until analysis. Platelet-rich plasma to be used in functional studies was prepared as described below.

### 2.5. Outcomes

The primary outcome measure was the effect of soffritto intake on vascular endothelial function and platelet activity (arachidonic acid, collagen, and adenosine diphosphate (ADP)). The secondary outcome measures were anthropometric measurements, blood pressure, lipid profile, hepatic and renal markers, and blood parameters.

#### 2.5.1. Platelet Aggregation

The study of platelet function was performed by analyzing platelet aggregation induced by different agonists: arachidonic acid (1 mM), collagen (2 and 5 µM), and ADP (5 and 20 µM) by the light transmission technique LTA (“Light Transmission Aggregometry”) in platelet-rich plasma prepared within an interval of 30 min from blood withdrawal at the different time-points of the study. The preparation contained 250,000 platelets/µL of platelet-poor plasma from each individual that was used as a 100% light transmission control. The recording of platelet aggregation was performed for a period of 5 min and was used for the calculation of the maximum aggregation [25]. Results after interventions are expressed as the percentage of variation with respect to the baseline value (100%).

#### 2.5.2. Flow Cytometric Analysis of Circulating Extracellular Vesicles

Three-label flow cytometry analysis was assessed as previously described by Suades et al. [26]. Briefly, PBS buffer containing 2.5 mmol of CaCl_2_ (Annexin Binding Buffer; ABB, BD Biosciences, San Jose, CA, USA) was used to dilute washed cEV suspensions. Afterwards, combinations of CFBlue-conjugated annexin V (AV) (Immunostep, Salamanca, Spain) with two specific monoclonal antibodies (mAb) labeled with fluorescein isothiocyanate (FITC) and phycoerythrin (PE) or the isotype-matched control mAb were added (Supplemental Table S1). Samples were incubated at room temperature in the dark for 20 min. Finally, samples were diluted with ABB before being immediately analyzed on a FACSCanto II™ (BD, Franklin Lakes, NJ, USA) flow cytometer.

Acquisition was performed at 1 min per sample at a low flow rate. Forward scatter (FSC), side scatter (SSC), and fluorescence data were acquired using settings configured on the logarithmic scale. Gate limits were established following the criteria previously described [26]. The upper threshold of 1 µm for FSC was set with the Flow Check YG Size Range Calibration (Polysciences, Warrington, PA, USA) with beads (1 µm diameter) and with Megamix-Plus FSC beads (mix of the following bead-equivalent diameters: 0.1, 0.3, 0.5, and 0.9 µm, BioCytex, Marseille, France). Based on the signal from the beads, the lower detection limit was established by setting a threshold above the electronic background noise of the flow cytometer for FSC and at the second logarithm for SSC. cEVs within the established gate limits (>0.1 to 1 µm) were identified and quantified based on their binding to AV and reactivity to cell-specific mAb. To identify positively marked events, fluorescence thresholds were similarly determined using samples that underwent titration experiments with the final concentration of an isotype-matched control mAb. AV binding level was corrected for auto-fluorescence using fluorescence signals obtained with cEVs in a calcium-free buffer (PBS). In order to minimize background noise, buffers were freshly prepared and then filtered through 0.2 µm pore-size filters using a vacuum system.

Data was analyzed with the BD FACSDiva™ Software (version 6.1.3). The concentration (number of cEVs per µL of plasma) was determined according to Nieuwland’s procedure [27], based on the sample’s volume, the flow cytometer’s flow rate, and the number of fluorescence-positive events (N), as shown in Equation (1):

(1) $$\frac{cMVs}{µL}=N\times \left(\frac{Vf}{Va}\right)\times \left(\frac{Vt}{FR}\right)\times \left(\frac{1}{VI}\right)$$

where *Vf* (µL) is the final volume of washed cEVs suspension; *Va* (µL) is the volume of washed cEVs suspension before fluorescence-activated cell sorting analysis; *FR* (µL/min) is the flow rate of the cytometer at low mode (the average volume of cEVs suspension analyzed in 1 min); 1 is the µL unit of volume; and *VI* (µL) is the original volume of plasma used for cEVs isolation.

#### 2.5.3. Anthropometric Data, Blood Pressure, Lipid Profile, and Other Biochemical Measurements

Trained personnel performed the anthropometric and clinical measurements (height, weight, waist circumference [WC], and blood pressure). Anthropometric and blood pressure measurements were determined at baseline and at the end of the treatment, before the extraction of the blood sample. BMI was obtained by dividing the body weight in kilograms by the square of height in meters (kg/m^2^). WC was measured between the lowest rib and the iliac crest with the participant standing. The waist-to-height ratio (WHtR) was obtained by dividing the waist circumference by the height, both measured in the same units (cm/cm).

Routine commercially available assays were utilized for the assessment of serum biochemical measurements, encompassing glucose levels, hepatic and renal markers, hemograms, and the standard serum lipid profile. including triglycerides (TAG), total cholesterol, and high-density lipoprotein cholesterol (HDLc) (Roche Diagnostics, Basel, Switzerland). As there were no cases of hypertriglyceridemia, low-density lipoprotein cholesterol (LDLc) was calculated using the Friedewald equation. Glomerular filtration rate was obtained according to the CKD-EPI Levey equation [28].

#### 2.5.4. Vascular Endothelial Function and Hemogram Profile

Endothelial function and blood parameters were assessed by digital plethysmography using the EndoPAT2000 device (Itamar Medical Ltd., Caesarea, Israel). Measurements were conducted following the guidelines provided by the manufacturer, with participants in a supine position, hands positioned at the same level, in a comfortable and thermoneutral environment at a temperature ranging from 21 to 24 °C. Arterial systolic and diastolic blood pressures were measured before starting the test. Pneumatic probes were placed on each index finger and a blood pressure cuff on one arm (study arm), while the contralateral arm served as a control (control arm). After a 10-min equilibration period, the blood pressure cuff on the study arm was inflated to 60 mmHg above systolic pressure for 5 min. The cuff was then deflated to induce reactive hyperemia, and the signals from both PAT channels (Probe 1 and Probe 2) were recorded by a computer. Occlusion was confirmed by the complete attenuation of the PAT signal from the test arm. To correct for systemic changes in vascular tone, recordings from the non-occluded arm (internal control) were used. The EndoPAT software package (version 3.4.4) was used to calculate endothelial function and arterial stiffness. Endothelial function was given as the reactive hyperemia index (RHI) and arterial stiffness as the augmentation index (AI), with AI standardized to a pulse of 75/min (AI@75) [29]. The natural logarithmically transformed RHI (lnRHI) values were also calculated. The Framingham RHI (FRHI) was calculated as the natural log-transformation of the RHI [30].

### 2.6. Statistical Analysis

Statistical analyses were conducted using STATA 15 (College Station, TX, USA) software. The data are expressed as the mean and the standard error of the mean. The normality of the distribution was assessed using the Shapiro–Wilk test. To assess statistical baseline differences between groups, a paired Student’s *t*-test was performed. To assess statistical differences in the changes observed at the end of the intervention, a paired Student’s *t*-test was performed. The effects of the six-week supplementation were evaluated using a paired Student’s t-test (baseline and post-intervention values) in each group. Correlations between age, BMI, or sex and the changes observed for platelet function, endothelial function, and blood lipid biomarkers were assessed by means of Pearson’s correlation. All reported *p*-values are two-sided, and values of 0.050 or below were considered statistically significant.

## 3. Results

The prospective, two-arm longitudinal crossover design, including enrollment, the randomization scheme, and final sample distributions by treatment group, are presented in the Consolidated Standards of Reporting Trials (CONSORT) diagram (Supplementary Figure S1).

The study was carried out in a group of 40 individuals (13 women and 27 men) with an average age of 40.8 ± 1.3 years. The entire study population completed both treatment phases, and no adverse effects were observed during the consumption of the tomato products. Adherence to the study dietary pattern was >97% during the 6 weeks of the intervention period. During the duration of the study, the volunteers were asked to maintain their usual diet, excluding raw or cooked tomatoes as well as tomato-based products (sauces, ketchup, juices, etc.) other than those administered during the study in the corresponding periods. Eighty-five percent of the individuals declared to have followed the indicated instructions, and 15% reported to have taken sporadically tomato or ketchup (4–8% of the days) within the usual range of a daily diet, irrespectively of the intervention stage (wash-out period, control, or soffritto intervention period). Therefore, all the individuals were included in the statistical study. 

### 3.1. Platelet Function

**Figure 2:**
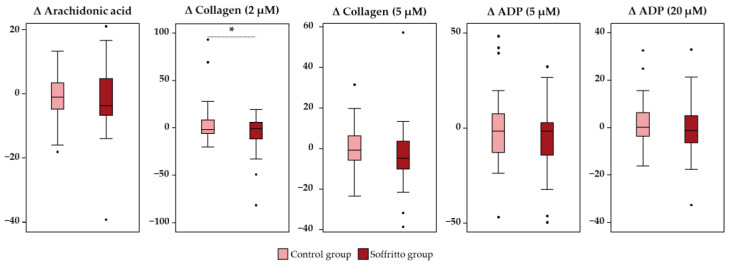
Differences in the change of platelet function between groups after six weeks of intervention (soffritto and control). * indicates a significant statistical difference (*p* < 0.05). Differences by group were analyzed by a paired Student’s *t*-test. *n* = 40.

As for platelet activity, the comparison between changes (effect of intervention—baseline) observed in the soffritto and control groups is presented in Figure 2. A marked tendency to decrease arachidonic acid-induced platelet aggregation (−2.26 ± 1.74%), collagen-induced platelet aggregation (5 µM) (−3.21 ± 2.41%), ADP-induced platelet aggregation (5 µM) (−4.61 ± 2.67%), and ADP-induced platelet aggregation (20 µM) (−3.67 ± 1.68%) was observed in the soffritto group without reaching significance (*p* > 0.05). Regarding collagen-induced platelet aggregation at 2 µM, a significant reduction was observed in the soffritto group in comparison to the control group (−5.10 ± 3.06% vs. 4.47 ± 3.52%, respectively) (*p* = 0.043) (Figure 2).

**Figure 3:**
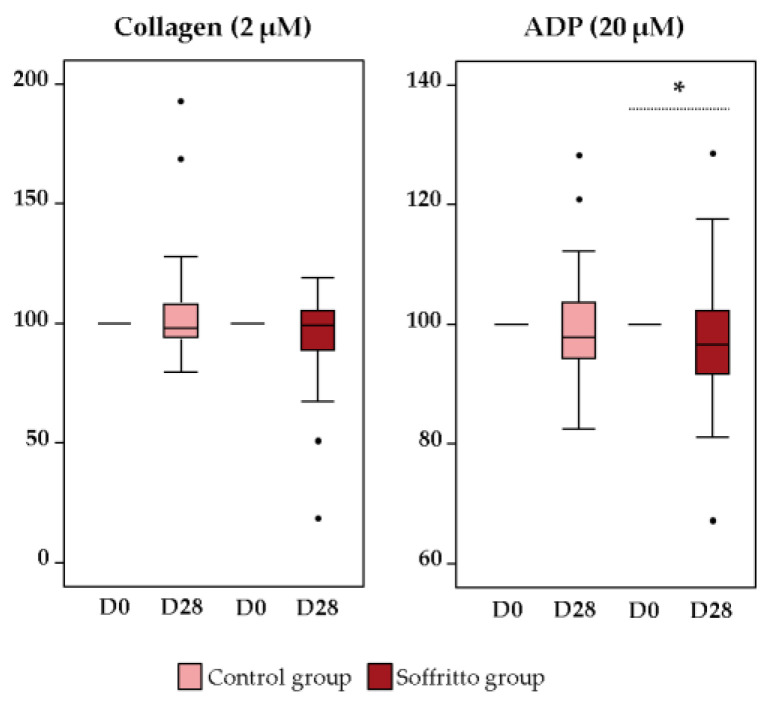
Comparison between baseline and final values for collagen-induced platelet aggregation (2 µM) and ADP-induced platelet aggregation (20 µM) after six weeks. * Indicates a significant statistical difference (*p* < 0.05). Differences by group were analyzed by a paired Student’s *t*-test. *n* = 40.

Given the significant decrease in collagen-induced platelet aggregation, the differences between baseline and final levels were analyzed; however, no statistically significant difference was observed (*p* > 0.05) (Figure 3). For the remaining variables, the differences between the baseline and the end were also assessed. A significant reduction was only observed for ADP-induced platelet aggregation at 20 µM in the soffritto group (*p* = 0.0352) (Figure 3). Regarding the control group, no significant differences were observed between the basal and final values (*p* > 0.05). In addition, the effect of daily soffritto intake on the different agonists was studied considering age, BMI, or sex (Spearman correlation), but no statistically significant differences were observed (Figure S2, Supplementary Materials).

### 3.2. Endothelial Function

Regarding endothelial function (RHI, lnRHI, FRHI, and AI@75), the analysis of baseline characteristics indicated that both intervention groups presented a similar endothelial function (*p* > 0.05) (Table 2).

The analysis of the differences observed between the beginning and the end of each of the interventions showed that the effect was similar in both groups in all the parameters analyzed (*p* > 0.05). However, it is important to note that, although not significant, the AI@75 tends to increase in the control group (2.49 ± 1.46), while a slight decrease was observed in the soffritto group (−0.21 ± 1.45). Additionally, the effect of the daily intake of Mediterranean soffritto on endothelial function was studied considering age, BMI, or sex (Spearman correlation), but no statistically significant differences were observed (Figure S2, Supplemental Materials).

cEVs carrying E-selectin (CD62E) and the cell adhesion molecule MUC-18 (CD146), released by activated endothelium, are found in very low quantities in comparison to the total amount of cEVs found in our study population (<0.5% of total cEV number) (Supplemental Material, Table S2). The released cEVs CD62E^+^ and CD146^+^ were not negatively affected by soffritto intake (blank without intervention). 

### 3.3. Anthropometric and Biochemical Variables

Baseline characteristics of the population after run-in (period 1) and wash-out (period 2) are depicted in Table 3. The subjects in the control group had a BMI of 31.16 ± 0.56 kg/m^2^, while those in the soffritto group had a BMI of 31.05 ± 0.51 kg/m^2^, without significant differences between groups (*p* > 0.05). In addition, a similar profile for weight, WC, and WtHR was observed between both groups. The groups were characterized by having normal values of SBP (<130 mmHg) and DBP (<85 mmHg), with no statistical differences between them (*p* > 0.05). Additionally, no differences in total protein, glucose, creatinine, urea, uric acid, ALT, AST, or GGT between the control group and the soffritto group were found (*p* > 0.05). 

The effect of a six-week soffritto intake is presented in Table 3. Regarding anthropometric parameters (weight, BMI, WC, and WtHR), the differences observed between the baseline and final values are similar in both groups (*p* > 0.05). Regarding hemodynamic parameters, the effect of the treatment was similar in both groups for systolic blood pressure. As for diastolic pressure, a significant decrease (−2.83 ± 1.28 mmHg) was observed in the control group (*p* = 0.040). However, despite the improvement in the control group, the participants who received the soffritto had pressure in the normal range. The differences in glucose and total protein were similar in both groups (*p* > 0.05). Kidney function (creatinine, urea, and uric acid) remained unaltered and within their normal range (*p* > 0.05). A similar behavior was observed for hepatic enzymes (ALT, AST, and GGT).

### 3.4. Lipid Profile

Analysis of the baseline blood lipid profile characteristics revealed that both groups presented a similar profile in TC, HDLc, LDLc, non-HDLc, LDLc/HDLc ratio, and TAG (Table 3) (*p* > 0.05).

The daily intake of soffritto preparation during a period of six weeks did not induce any significant changes in the levels of TC, TAG, and cholesterol transported by HDL, LDL, and non-LDL (*p* > 0.05) in overweight and obese individuals (Table 3). A similar behavior was observed in the control group. In addition, no changes were observed in the relationship between LDLc and HDLc (*p* > 0.05) in both groups. The most obvious, although not significant, difference was with the level of TAG (−8.91 ± 11.50 vs. 7.76 ± 8.40 mg/dL, control and soffritto, respectively). However, the increase in TAG in the soffritto group was not significant.

Furthermore, the impact of daily consumption of soffritto on blood lipids was examined, considering factors such as age, BMI, or sex (analyzed through Spearman correlation). However, no statistically significant differences were noted, as depicted in Figure S2 of the Supplementary Material.

### 3.5. Blood Parameters

Regarding hemoglobin, red and white blood cells, and platelets, no statistically significant differences were observed between the two groups at baseline (*p* > 0.05) (Supplemental Table S3). Similar behavior was observed for mean corpuscular hemoglobin concentration, red blood cell distribution width, mean corpuscular volume, and the combined value of the other types of white blood cells not classified as lymphocytes or granulocytes (*p* > 0.05). 

Analysis of the differences observed after six weeks of daily intake of soffritto or in the control group showed that there were no statistically significant differences between the two groups in all the variables previously mentioned after six weeks (*p* > 0.05).

## 4. Discussion

The beneficial effect of fresh tomatoes or their derivatives (e.g., purée, sauces, ketchup) in reducing conventional CVD risk markers has been deeply studied in the last few decades [14,31]. Nevertheless, based on our current understanding, little is known about the effect of soffritto, a basic culinary technique widely used in the Mediterranean area rich in carotenoids and polyphenols [23], on the overweight and obese population. In this study, we conducted a single-center, prospective, controlled, randomized, two-arm longitudinal crossover trial. We explored the effects of daily soffritto intake during six weeks in overweight or obese class 1 individuals without other cardiovascular risk factors. Our main focus was on the effect of soffritto intake on platelet activity. Additionally, any undesirable changes associated with the daily intake of soffritto in vascular endothelial function, body weight changes, lipoprotein profile, and arterial stiffness were also assessed.

Regarding our main outcome, platelet activity, so far it is well known that platelet activation is triggered by several intracellular signaling cascades stimulated by different soluble agonists such as ADP, collagen, and arachidonic acid, among others. Collagen induces platelet aggregation by triggering granule secretion through glycoprotein VI (GPVI) and proteinase-activated receptor signaling. ADP induces platelet activation that mediates the P2Y12 or P2Y1 receptor-signaling pathway [32]. Arachidonic acid is released by activation of soluble phospholipase A2 and metabolized by cyclooxygenases (COX) and lipoxygenases (LOX) [22]. Regarding ADP-induced platelet aggregation, in our study, the findings from the soffritto group after a 6-week intervention suggest a trend towards reduction (at 5 and 20 µM), although statistical significance was not achieved. However, the differences between baseline and final levels revealed a significant decrease in ADP-induced platelet aggregation at 20 µM. With respect to collagen-induced platelet aggregation, inhibition was observed in the volunteers that received soffritto (differences between groups). The fact that soffritto supplementation reduced collagen- and ADP-induced LTAs whereas it did not affect arachidonic acid-induced platelet aggregation likely reflects the ability of soffritto to differentially interfere with the multiple signaling cascades involved in platelet activation. As such, our findings suggest that soffritto may interfere with the interaction of collagen and ADP with platelet receptors (mainly GPVI and P2Y12) and consequently their downstream platelet aggregation-triggering pathways (PLCbeta/PKC and PLCgamma/PKC). In contrast, the lack of a statistically significant decrease in arachidonic-acid-induced platelet aggregation suggests that the soffritto effects were not mediated via intraplatelet COX-1 and thromboxane synthase [33,34]. Further studies are needed to elucidate the exact mechanisms by which soffritto modulates the platelet response.

To date, scientific evidence of the beneficial effects of tomato-base products on CVD and platelet aggregation has been demonstrated in in vitro [18,35,36,37] and in vivo [17,35] studies. In a double-blinded crossover study in healthy humans, it was evidenced that the consumption of tomato supplements in drink format led to a significant reduction in ex vivo platelet aggregation after 3 h [18]. In another study, it was found that a 150 mg/day water-soluble tomato extract supplement for four weeks significantly reduced ADP-induced or collagen-induced platelet aggregation in healthy middle-aged and older individuals [17]. The favorable impact of fresh tomatoes or tomato-base products on platelet aggregation is contingent upon their adequate presence of bioactive compounds (such as lycopene, flavonoids, phenolic acids, and vitamin E) [38]. In an in vitro study, it was demonstrated that lycopene (2–12 mmol/L) concentration-dependently inhibited platelet aggregation in human platelets and the ATP-release reaction stimulated by agonists (arachidonic acid and collagen) [36]. Regarding phenolic compounds, an in vitro study also evidenced that *p*-coumaric, chlorogenic, ferulic, and caffeic acids possess antiplatelet activity [37]. As a notable finding, our study revealed that soffritto may have a role in the primary prevention of CVD by reducing platelet activation, independent of sex, age, or BMI. In other words, the trend was to improve the entire population.

Based on our obtained results, it can be extrapolated that the consumption of soffritto among overweight and obese individuals does not exhibit any discernible negative influence on endothelial function, as demonstrated by measuring the vascular resistance to hyperemic conditions and the release of eEVs containing markers of activated endothelial cells. This is important, as endothelial function is one of the most reliable markers of vascular health, and its deterioration is considered the common pathway linking coronary risk factors to the progression of atherosclerosis [39,40]. As of the current date, the evidence pertaining to the impact of tomato products, or their bioactive compounds has generated divergent findings. In the study carried out by Stangl et al. [41], it was demonstrated that both acute (24 h) and long-term (7 days) tomato purée consumption had no effects on endothelium-dependent or endothelium-independent dilation of the brachial artery in healthy postmenopausal women. Midterm (15 days) tomato paste dietary supplementation (70 g tomato paste—33.3 mg of lycopene) improved endothelial functions in healthy young women and men; however, these findings were not observed after acute ingestion [42]. On the other hand, daily lycopene intake (15 mg/day) for eight weeks in healthy men significantly improved endothelial function, mainly in those subjects with relatively impaired endothelial cell function at the initial level [43]. In one study, it was proposed that the beneficial effect of tomato or tomato-based product consumption is conditioned by its processing [31]. In the study, it was observed that tomato sauce fortified with olive oil exerts a more pronounced impact on CVD risk factors compared to both raw tomato and regular tomato sauce. In hypercholesterolemic pigs, the daily intake of soffritto was associated with protection against LDL-induced coronary endothelial dysfunction by reducing oxidative damage, enhancing eNOS expression and activity, and improving HDL functionality [44]. Therefore, the observed disparities in the impact of tomato product consumption on endothelial function may be attributed to the matrix (purée, paste, soffritto) or comorbidities of the host, which could influence the bioavailability of bioactive compounds, thereby affecting their efficacy [45].

Another of the findings of our study was that the daily intake of soffritto during the six weeks did not affect any of the anthropometric variables or biochemical profiles of overweight and obese individuals. In a study carried out in 2013, four weeks of regular consumption of high-lycopene sauce or commercial sauce did not induce changes in anthropometrical and biochemical variables (weight, BMI, WC, insulin, glucose, and uric acid) in a healthy population [46]. A similar trend was observed after four weeks of daily intake of two fresh tomatoes in individuals with low HDLc (men and women < 40 and < 50 mg/dL, respectively) but normal TAG levels (<150 mg/dL) [47]. In our study, it is important to note that although an increase in TAG was observed in the soffritto group, this was not significant and could be due to the composition of the soffritto (5% of extra virgin olive oil). Regarding the tomato soffritto effect, in the study carried out by Hurtado-Barroso et al. [48], an improvement in urea, total proteins, and albumin was observed after 24 h. However, creatinine and uric acid remained unchanged. Additionally, twelve weeks of a high-tomato-based diet or a diet supplemented with lycopene capsules also did not improve the glucose and insulin levels in healthy middle-aged volunteers [14].

One additional finding in our study is that the daily intake of soffritto preparation does not induce changes in the serum lipid profile in individuals with overweight or obesity. In the study carried out by García-Alonso et al. (2012) [15], it was evidenced that intervention with tomato juice and ω3-enriched tomato juice for 2 weeks failed to ameliorate the blood lipids of healthy women. Another six-week randomized controlled trial demonstrated that a significant daily intake of tomato-based products (equivalent to 32–50 mg lycopene/day) or lycopene supplements (10 mg/day) does not effectively reduce conventional CVD risk markers, including lipid profile, in moderately overweight, healthy, middle-aged individuals [14]. In addition, a systematic review and meta-analysis demonstrated that tomato products and lycopene supplementation only result in a reduction of LDLc but not in other blood lipids (TC, HDLc, and TAG) [49]. Short intervention periods [15], low intake of tomato-based foods [14], and the health status of the study population [14] stand out as the main reasons for this lack of effect on blood lipids. Not surprisingly, no differences in blood parameters were observed since this is in line with previous studies. In the study carried out by García-Alonso et al. [15], two-weeks of daily intake of tomato juice or ω3-enriched tomato juice (500 mL, 181 mg polyphenols, and 26.5 mg of lycopene) did not induce changes in RBC, hemoglobin, hematocrit, WBC, and platelet in healthy women. The same behavior was observed in the study carried out by Nishimura et al. [16], where no changes were observed for RBC, WBC, hemoglobin, hematocrit, and platelets after twelve weeks of tomato intake. 

This study faced several limitations, primarily related to the sample size. Consequently, further investigation with a larger sample size is needed to validate the vascular and platelet effects associated with the intake of soffritto in overweight and obese individuals. Another of the limitations of our study lies in the fact that the participants enrolled in this trial, despite being overweight or obese, may have been in generally good health, which could have made it difficult to identify significant changes in CVD risk markers. Therefore, the inclusion of volunteers with CVD risk factors and/or a higher BMI (obesity class II) could have increased the likelihood of detecting changes.

## 5. Conclusions

The remarkable health benefits of tomatoes are widely acknowledged, boasting an impressive array of properties due to their antioxidants, vitamins, glycoalkaloids, and minerals. Surprisingly, although soffritto is a staple in the Mediterranean diet, its potential health effects remain largely uncharted territory in scientific research. Soffritto, being cooked tomatoes, has increased antioxidant properties, but whether its additional components had any unwanted effects was unknown.

The present study suggests that the consumption of soffritto resulted in a reduction in platelet aggregation after a six-week period in overweight and obese subjects. The observed effects were more evident in collagen and ADP-induced platelet aggregation. No significant effects of the soffritto intake were observed for anthropometric measures, serum lipid profile, vascular endothelial function, or arterial stiffness. We conclude that the intake of tomato soffritto in overweight and obese subjects is safe and may have a role in the primary prevention of CVD by reducing platelet activation, which could contribute to a reduction in thrombotic events.

## Figures and Tables

**Table 1:** Soffritto composition.

**Ingredients of the Soffritto (%)**	
Tomato paste	35
Onion	10
Extra-virgin olive oil	5
Sugar	3
Salt	2
**Mean Nutritional Values (100 g)**	
Energetic value	96 Kcal/400 KJ
Proteins	1.8 g
Carbohydrates	10.6 g
Fat	5.1 g

**Table 2:** Endothelial function at baseline and changes observed after 6 weeks in the control group and in the soffritto group.

	Baseline Characteristics			∆		
	Control	Soffritto	*p*-Value	Control	Soffritto	*p*-Value
RHI	2.01 ± 0.13	1.85 ± 0.06	0.106	−0.02 ± 0.13	0.12 ± 0.09	0.364
lnRHI	0.64 ± 0.05	0.60 ± 0.03	0.320	0.00 ± 0.05	0.04 ± 0.04	0.526
FRHI	0.35 ± 0.07	0.30 ± 0.05	0.306	−0.03 ± 0.06	0.01 ± 0.04	0.533
AI@75	−1.27 ± 1.85	−0.39 ± 2.10	0.619	2.49 ± 1.46	−0.21 ± 1.45	0.270

RHI: reactive hyperemia index; lnRHI: logarithm of RHI; FRHI: Framingham RHI; AI@75: augmentation index standardized to a pulse of 75/min. Data are expressed as mean ± SEM. Differences at baseline and in the effect of treatment between groups were analyzed by a paired Student’s *t*-test. *n* = 40; *p* < 0.05 indicates significance.

**Table 3:** Obesity-related variables and serum lipid levels at baseline and changes after a 6-week dietary intervention without (control) or with soffritto.

	Baseline Characteristics			∆		
	Control	Soffritto	*p*-Value	Control	Soffritto	*p*-Value
***Anthropometric parameters***						
Weight (kg)	90.00 ± 2.31	89.78 ± 2.12	0.110	−0.38 ± 0.29	−0.08 ± 0.24	0.401
BMI (kg/m^2^)	31.16 ± 0.56	31.05 ± 0.51	0.481	−0.13 ± 0.13	0.03 ± 0.09	0.386
WC (cm)	100.53 ± 1.86	100.86 ± 1.78	1.000	−0.78 ± 0.78	−0.98 ± 0.80	0.324
WtHR (cm/cm)	0.59 ± 0.01	0.60 ± 0.01	1.000	0.00 ± 0.00	−0.01 ± 0.00	0.221
***Hemodynamic control***						
SBP (mmHg)	125.79 ± 2.20	124.35 ± 1.78	0.362	−2.50 ± 1.80	−0.23 ± 1.37	0.341
DBP (mmHg)	72.59 ± 1.95	70.78 ± 1.66	0.172	−2.83 ± 1.28	0.68 ± 1.18	0.040
**Biochemical parameters**						
Total protein (g/L)	68.82 ± 0.60	66.93 ± 1.68	0.219	−1.21 ± 0.47	0.79 ± 1.90	0.301
Glucose (mM)	4.91 ± 0.14	4.83 ± 0.13	0.329	−0.10 ± 0.06	−0.09 ± 0.07	0.981
Creatinine (µM)	76.23 ± 1.79	75.70 ± 1.67	0.460	−0.39 ± 0.72	0.80 ± 0.92	0.256
Urea (mM)	5.48 ± 0.19	5.22 ± 0.17	0.151	0.19 ± 0.17	0.22 ± 0.19	0.914
Uric acid (mM)	351.00 ± 16.70	349.35 ± 16.16	0.603	14.95 ± 6.75	3.73 ± 9.65	0.284
ALT (U/L)	25.03 ± 3.03	22.90 ± 2.20	0.200	−4.88 ± 3.22	−0.45 ± 2.40	0.326
AST (U/L)	22.46 ± 2.64	22.05 ± 1.22	0.873	−0.15 ± 1.76	−0.15 ± 1.51	1.000
GGT (U/L)	31.87 ± 4.76	30.28 ± 4.41	0.242	1.78 ± 1.48	1.93 ± 1.53	0.920
**Lipid profile**						
TC (mg/dL)	194.87 ± 4.76	193.84 ± 5.27	0.347	−1.03 ± 3.02	1.54 ± 2.08	0.468
HDLc (mg/dL)	46.40 ± 1.79	45.80 ± 1.56	0.621	−0.15 ± 0.82	0.27 ± 0.69	0.728
LDLc (mg/dL)	116.41 ± 4.26	118.91 ± 4.22	0.484	0.54 ± 3.00	−0.97 ± 1.96	0.678
Non-HDLc (mg/dL)	148.47 ± 4.88	146.03 ± 5.30	0.369	−0.88 ± 2.64	1.27 ± 2.05	0.494
LDLc/HDLc	2.60 ± 0.12	2.69 ± 0.13	0.239	0.03 ± 0.06	−0.08 ± 0.05	0.170
TAG (mg/dL)	159.28 ± 21.22	131.26 ± 13.27	0.115	−8.91 ± 11.50	7.76 ± 8.40	0.265

ALT: alanine transaminase; AST: aspartate transaminase; BMI: body mass index; DBP: diastolic blood pressure; GGT: gamma-glutamyltransferase; SBP: systolic blood pressure; TAG: triacylglycerols; TC: total cholesterol; WC: waist circumference; WtHR: waist-to-height ratio. Data are expressed as mean ± SEM. Differences in the baseline values between groups were analyzed by a paired Student’s *t*-test. Differences in the effect of treatment between both groups were analyzed by a paired Student’s *t*-test. *n* = 40; *p* < 0.05 indicates significance.

## Data Availability

The information outlined in the manuscript, as well as the code book and analytic code, will be provided upon a reasonable request, subject to scientific approval.

## Supplementary Materials

The following supporting information can be downloaded at: https://www.mdpi.com/article/10.3390/nu15245084/s1, Figure S1: CONSORT diagram; Figure S2: Spearman correlation between age, BMI and sex with the changes observed for platelet function, endothelial function and blood lipids biomarkers in subjects who received soffrito or in the control group; Table S1: Cell surface molecules for circulating microparticles identification and characterization; Table S2: Circulating levels of endothelial extracellular vesicles (cEVs) at baseline and at the end of the control and soffritto intervention periods; Table S3: Hemogram profile at the beginning of the intervention and after six weeks of dietary intervention without (control) or with soffritto.
