# Supplementary material for: Vascular and Platelet Effects of Tomato Soffritto Intake in Overweight and Obese Subjects
Source: Nutrients. 2023 Dec 12;15(24):5084. doi: 10.3390/nu15245084 (PMC10745891; doi:10.3390/nu15245084)
Supplement: Supplementary file 1 [file nutrients-15-05084-s001.zip › nutrients-2672502-supplementary.pdf]

Vascular and Platelet Effects of Tomato Soffritto Intake in Overweight and Obese Subjects

Anallely López-Yerena [1,†], Teresa Padro [1,2,†], Victoria de Santisteban Villaplana [1,3], Natàlia Muñoz-García [1], Antonio Pérez [4,5], Gemma Vilahur [1,2] and Lina Badimon [1,2,6,*]

[1] Cardiovascular Program-ICCC, Institut d'Investigació Biomèdica Sant Pau (IIB SANT PAU), 08041 Barcelona, Spain; naye.yerena@gmail.com (A.L.-Y.); tpadro@santpau.cat (T.P.); vsantisteban@santpau.cat (V.d.S.V.); nmunoz@santpau.cat (N.M.-G.); gvilahur@santpau.cat (G.V.)
[2] Centro de Investigación Biomédica en Red Cardiovascular (CIBER-CV), Instituto de Salud Carlos III, 28029 Madrid, Spain
[3] Faculty of Pharmacy, University of Barcelona, 08036 Barcelona, Spain
[4] Servicio de Endocrinología y Nutrición, Hospital de la Santa Creu i Sant Pau, IIB Sant Pau, Universitat Autònoma de Barcelona, 08041 Barcelona, Spain; aperez@santpau.cat
[5] CIBER de Diabetes y Enfermedades Metabólicas Asociadas (CIBERDEM), 08041 Barcelona, Spain
[6] Cardiovascular Research Chair, Universitat Autònoma de Barcelona (UAB), 08193 Barcelona, Spain
* Correspondence: lbadimon@santpau.cat
† These authors contributed equally to this work.

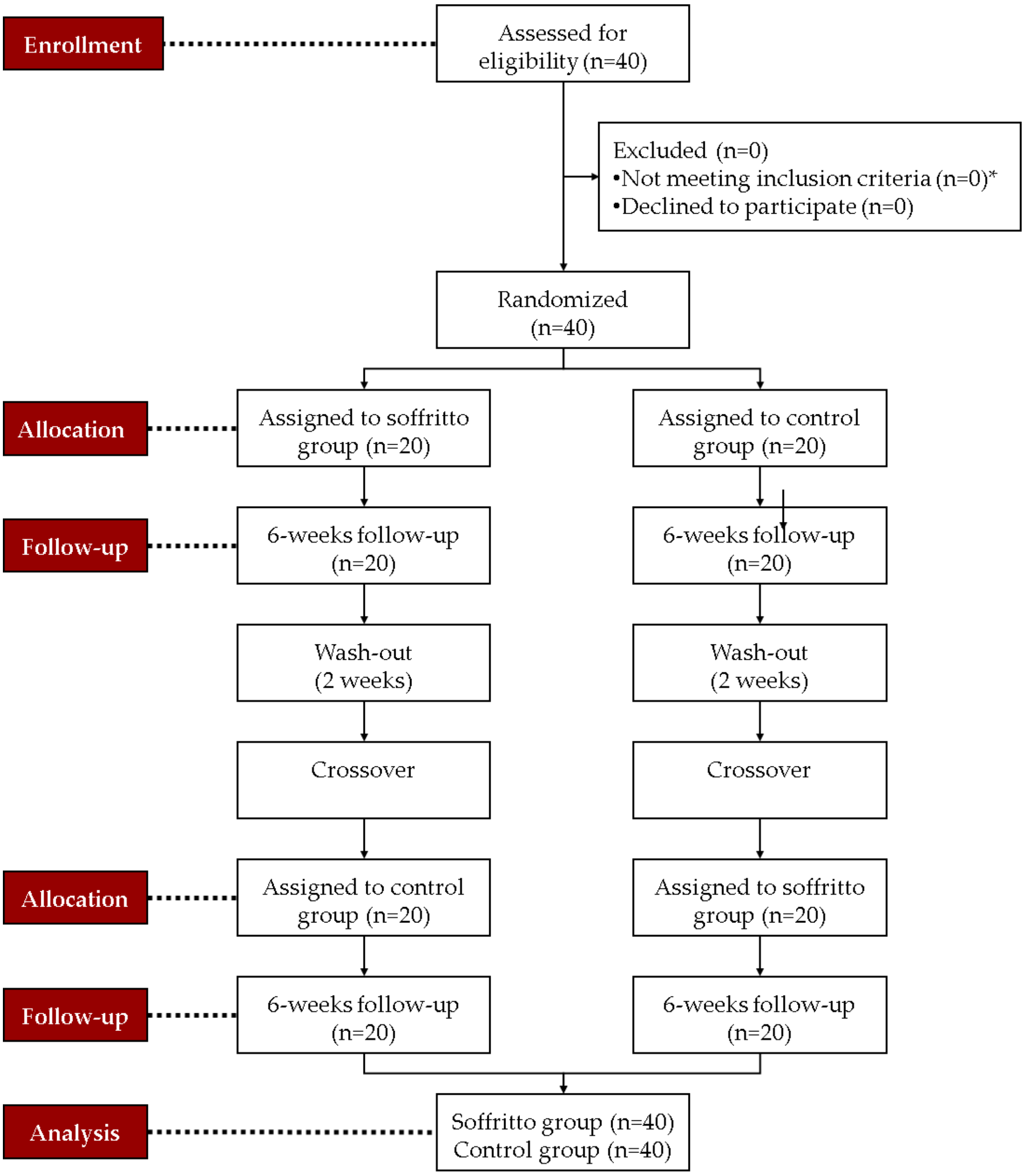

Figure S1. CONSORT diagram. *Subjects were excluded if they reported existing chronic illnesses including cancer, dyslipidaemia, diabetes mellitus, hypertension, heart, liver or kidney disease (creatinine > 2 mg/dL) and excessive alcohol consumption (> 60 g day of ethanol). Other exclusion criteria included the use of lipid-lowering drugs, β-blockers or diuretics, history of CVD, psychiatric illness or treatment of psychotropic drugs, being in a weight-loss program or intolerance to tomato-based products. In addition, subjects who reported taking aspirin in the 10-day period prior to blood sampling were excluded.

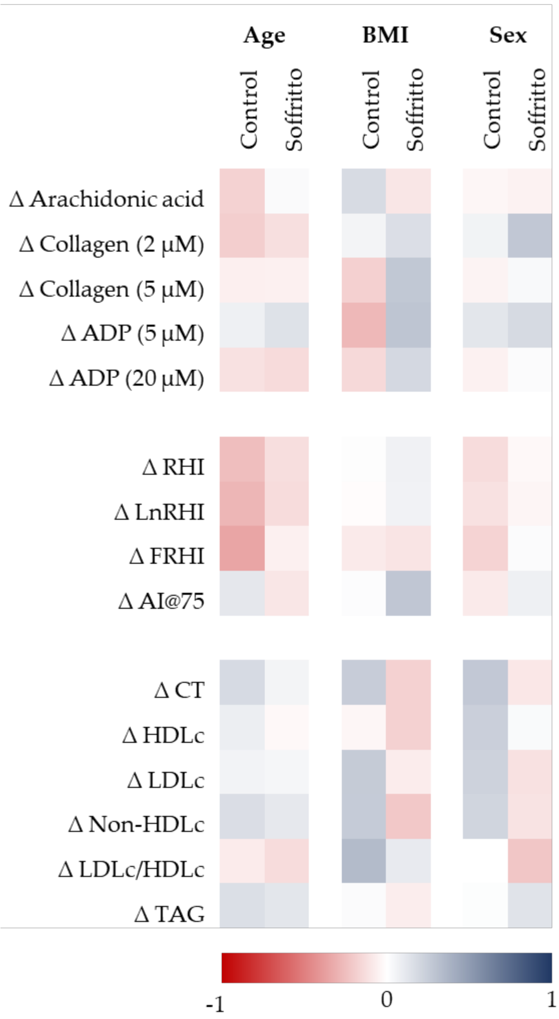

Figure S2. Spearman correlation between age, BMI and sex with the changes observed for platelet function, endothelial function and blood lipids biomarkers in subjects who received soffrito or in the control group. In this pairwise correlation, red shows a negative correlation and blue a positive correlation. ADP: adenosine diphosphate; RHI: reactive hyperaemia index; lnRHI: logarithm of RHI; FRHI: Framingham RHI; AI@75: augmentation index standardized to a pulse of 75/min; TC: Total cholesterol; TAG: Triglycerides. No significant correlations were observed in both groups.

Table S1. Cell surface molecules for circulating microparticles identification and characterization

	Marker	Expression	Alternative Name	Conjugation	Clone	Company
AV⁺-MEV	PS	Widely expressed	PS-binding protein	CF405	-	Immunostep
Endothelial markers	CD146⁺	Endothelial cells	MUC-18	FITC	P1H12	BD Pharmingen
	CD62E⁺	Activated endothelial cells	E-selectin	PE	68-5H11	

AV+-MEV: Microvesicles annexin V+; PE: Phycoerythrin; FITC: Fluorescein isothiocyanate; PS: phosphatylserine; MUC-18 (S-Endo 1): Cell adhesion molecule.

Table S2. Circulating levels of endothelial extracellular vesicles (cEVs) at baseline and at the end of the control and soffritto intervention periods

eEVs	Baseline Characteristics	Final Intervention		p-Value		
		Control	Soffritto	1	2	3
Total MVs	4554.71 ± 1223.19	3381.78 ± 761.77	3236.11 ± 517.60	0.80	0.88	0.45
Annexin V+	578.72 ± 58.62	617.74 ± 85.29	442.5 ± 45.28	0.64	0.57	0.16
CD146+	1.86 ± 0.37	1.77 ± 0.56	1.19 ± 0.38	0.53	0.72	0.36
CD146+/AV+	0.22 ± 0.09	0.27 ± 0.11	0.36 ± 0.14	0.53	0.32	0.79
CD62E+	15.11 ± 1.84	19.28 ± 3.31	17.51 ± 2.97	0.21	0.20	0.86
CD62E+/AV+	5.81 ± 0.83	9.44 ± 2.07	5.24 ± 0.96	0.54	0.80	0.42
CD62E+/CD146+	0.97 ± 0.19	0.65 ± 0.19	0.74 ± 0.25	0.43	0.32	0.83
CD62E+/CD146+/AV+	0.16 ± 0.09	0.15 ± 0.09	0.15 ± 0.09	0.53	0.08	1.00

Data (number cEVs/μL of plasma) are expressed as mean ± SEM. p-value[1]: Comparison between final and baseline values in the control group; p-value[2]: Comparison between final and baseline values in the soffritto group and p-value[3]: Comparison of final values between the control group and the soffritto group. Differences were analyzed by U Mann-Whitney-test. n = 39. $p < 0.05$ indicates significance.

Table S3. Hemogram profile at the beginning of the intervention and after six weeks of dietary intervention without (control) or with soffritto.

	Baseline Characteristics			Δ		
	Control	Soffritto	*p-Value*	Control	Soffritto	*p-Value*
Hemoglobin (g/dL)	11.76 ± 0.16	11.71 ± 0.15	0.225	-1.09 ± 0.38	-0.75 ± 0.12	0.394
RBC (10^6 mm)	4.35 ± 0.06	4.35 ± 0.06	0.685	-0.10 ± 0.03	-0.15 ± 0.04	0.292
Platelet (10^3 mm^3)	204.92 ± 6.82	212.15 ± 6.02	0.065	-1.53 ± 6.04	-14.45 ± 4.31	0.085
WBC (10^3 mm^3)	6.10 ± 0.24	6.10 ± 0.27	0.952	-0.11 ± 0.23	-0.08 ± 0.19	0.918
BLHR	66.82 ± 1.68	67.05 ± 1.61	0.749	-2.48 ± 1.80	-1.25 ± 1.24	0.593
Granulocyte (%)	49.71 ± 1.47	50.54 ± 1.00	0.558	-1.38 ± 1.73	-1.64 ± 1.38	0.910
Granulocyte (10^3 mm^3)	2.96 ± 0.14	3.11 ± 0.18	0.251	-0.06 ± 0.13	-0.10 ± 0.12	0.821
Hematocrit (%)	41.41 ± 4.54	36.91 ± 0.45	0.316	-6.36 ± 4.47	-0.87 ± 0.59	0.229
Lymphocytes (%)	42.83 ± 1.01	42.78 ± 1.01	0.938	-0.18 ± 1.35	0.45 ± 0.69	0.701
Lymphocytes (10^3 mm^3)	2.51 ± 0.09	2.55 ± 0.10	0.631	0.00 ± 0.09	0.02 ± 0.08	0.913
MCH (pg)	27.21 ± 0.25	26.98 ± 0.21	0.258	-1.80 ± 0.64	-0.86 ± 0.19	0.183
MCHC (g/dL)	31.15 ± 0.78	31.79 ± 0.14	0.413	0.00 ± 1.13	-0.98 ± 0.21	0.878
MCV (μm^3)	84.94 ± 0.49	83.21 ± 1.76	0.315	-2.42 ± 2.02	1.56 ± 1.63	0.123
MID (%)	6.80 ± 0.17	6.69 ± 0.14	0.489	-0.12 ± 0.29	0.07 ± 0.17	0.569
MID (10^3 mm^3)	1.28 ± 0.82	0.45 ± 0.02	0.319	-0.83 ± 0.80	-0.01 ± 0.02	0.315
MPV (μm^3)	8.24 ± 0.11	28.14 ± 19.84	0.323	-0.18 ± 0.23	0.25 ± 28.55	0.988
PRP-PLT	294.54 ± 11.30	304.65 ± 11.41	0.219	-22.45 ± 10.39	-10.35 ± 7.62	0.335
RDW (%)	17.39 ± 0.15	17.27 ± 0.15	0.508	-0.25 ± 0.44	0.30 ± 0.14	0.246

MCH: mean corpuscular hemoglobin; MCHC: Mean corpuscular hemoglobin concentration; MCV: mean corpuscular volume; MID: combined value of the other types of white blood cells not classified as lymphocytes or granulocytes; MPV: Mean platelet volume; PRP-PLT: Platelet rich plasma: Platelets; RBC: red blood cells; RDW: red blood cells distribution width; WBC: white blood cells. Data are expressed as mean ± SEM. Differences at baseline between groups were analyzed by paired Student's t -test. Differences in the changes observed between both groups were analyzed by paired Student's t -test. n = 40. $p < 0.05$ indicates significance.
